# Fluorescent microspheres can affect *in vitro* fibrinolytic outcomes

**DOI:** 10.1371/journal.pone.0284163

**Published:** 2023-04-07

**Authors:** Ethan G. Stoll, Sean J. Cone, Spencer R. Lynch, Andrew T. Fuquay, Brittany E. Bannish, Nathan E. Hudson

**Affiliations:** 1 Dept. Physics, East Carolina University, Greenville, North Carolina, United States of America; 2 Department of Biological Sciences, North Carolina State University, Raleigh, North Carolina, United States of America; 3 Medical Physics Graduate Program, Duke University, Durham, North Carolina, United States of America; 4 Dept. Mathematics and Statistics, University of Central Oklahoma, Edmond, Oklahoma, United States of America; Hamamatsu University School of Medicine, JAPAN

## Abstract

Hemostasis is the cessation of bleeding due to the formation of a blood clot. After the completion of wound healing, the blood clot is typically dissolved through the natural process of fibrinolysis, the enzymatic digestion by plasmin of the fibrin fibers that make up its structural scaffold. *In vitro* studies of fibrinolysis reveal mechanisms regulating these processes and often employ fluorescent microscopy to observe protein colocalization and fibrin digestion. In this study, we investigate the effects of labeling a fibrin network with 20 nm diameter fluorescent beads (fluorospheres) for the purpose of studying fibrinolysis. We observed fibers and 2-D fibrin networks labeled with fluorospheres during fibrinolysis. We found that the labeling of fibrin with fluorospheres can alter fibrinolytic mechanisms. In previous work, we showed that, during lysis, fibrin fibers are cleaved into two segments at a single location. Herein we demonstrate that fibrinolysis can be altered by the concentration of fluorospheres used to label the fibers, with high concentrations of fluorospheres leading to very minimal cleaving. Furthermore, fibers that are left uncleaved after the addition of plasmin often elongate, losing their inherent tension throughout the imaging process. Elongation was especially prominent among fibers that had bundled together due to other cleavage events and was dependent on the concentration of fluorophores used to label fibers. Of the fibers that do cleave, the site at which they cleave also shows a predictable trend dependent on fluorosphere concentration; low concentrations heavily favor cleavage locations at either end of fibrin fiber and high concentrations show no disparity between the fiber ends and other locations along the fiber. After the initial cleavage event bead concentration also affects further digestion, as higher bead concentrations exhibited a larger population of fibers that did not digest further. The results described in this paper indicate that fluorescent labeling strategies can impact fibrinolysis results.

## Introduction

Fibrinogen, an abundant plasma protein, constantly circulates in the vasculature at normal concentrations of 1.5–4.0 mg/mL [1]. Shortly after the onset of vascular injury, a cascade of enzymatic activation reactions culminates in the conversion of soluble fibrinogen into insoluble fibrin and the creation of a blood clot, which serves to temporarily stop or slow the flow of blood at the injury site [1]. Forming the structure of this blood clot is an insoluble fibrin network, which captures various cells and extracellular material [2]. Associated dangers and short life expectancy in individuals with a depressed ability to form sufficient clot, such as in dysfibrinogenemia or hemophilia, are evidence of the critical role clotting plays in the injury healing process [3].

Normally, plasmin, a proteolytic enzyme activated from its zymogen precursor, plasminogen, degrades the fibrin network after sufficient time for healing has passed, restoring normal blood flow around the injury site [4]. Hindered digestion of clots can lead to a myriad of pathologies, including myocardial infarction and ischemic stroke, two of the leading causes of death worldwide, both caused by the incomplete dissolution of thrombi [5].

Researching fibrinolysis and its complexities *in vivo* is technically difficult (requiring experiments on immobilized live animals) and leads to challenges in differentiating cause and effect due to the many molecular processes in fibrinolysis [6]. Because of this, many studies seeking to understand fibrinolytic mechanisms employ *in vitro* methodologies, with fluorescent microscopy being particularly popular [7]. Multiple variations of fluorophores or other labeling techniques have previously been utilized to study fibrin network structures using microscopy, including: colloidal gold particles which bind non-specifically to fibers and have been shown to not impede or alter fibrin network formation [8], fluorescent dyes such as fluorescein isothiocyanate (FITC) and Alexa-488 that are specifically bound to individual fibrin molecules [9, 10], fluorescently labeled antibodies [11], and carboxylate-coated beads, which are the subject of this investigation [12–15]. Each labeling method comes with associated benefits and drawbacks and relies on the addition of foreign chemicals and particles to a system. Because of this, it is important to understand any effects on experimental outcomes that a particular labeling method causes.

Here we focus on investigating the unexpected fibrinolytic effects of using 20 nm diameter, carboxylate-coated polystyrene beads to label fibrin fibers during fibrinolysis studies. The beads are commonly utilized in other fibrin studies due to their photostability and low photobleaching [16–18], and have recently been utilized in fibrinolysis studies [12–15]. However, in some studies of lysis utilizing these beads, fibers losing tension in a process referred to as elongation, rather than cleavage, were reported [12, 13]. Here, we investigated whether elongation could be a function of this fluorescent labeling approach, as opposed to other fibrinolytic mechanisms. In addition, we investigate mechanisms, such as fiber bundling, that lead to increased elongation. Our results along with others [15] indicate that at certain concentrations, fluorescent beads can alter fibrinolytic outcomes, and thus they should be used cautiously when employed in *in vitro* fibrinolytic studies.

## Materials and methods

### Sample preparation

On a glass microscope slide a small amount of optical glue (Norland Optical Adhesive 81, Norland Products, Cranbury, NJ), pressed under a PDMS stamp, was cured under ultraviolet light, resulting in a ridged surface with gaps between ridges measuring 20 μm wide and 10 μm deep. Aliquots of peak 1 fibrinogen (Enzyme Research Laboratories, South Bend, IN), ALEXA-488 fibrinogen (Invitrogen by Thermo Fisher Scientific/Life Technologies Corporation, Eugene, OR), and human alpha thrombin (Enzyme Research Laboratories, South Bend, IN) were thawed from -80°C. In a microcentrifuge tube, fibrinogen was diluted to 0.6 mg/mL with a HEPES Buffered Saline (HBS; 20 mM HEPES, 150 mM NaCl, pH 7.4) and mixed with ALEXA-488 fibrinogen at 1/65^th^ the fibrinogen concentration. Separately, the thrombin aliquot was diluted to 2 U/mL using HBS with CaCl_2_. 10 μL of fibrinogen solution was pipetted onto the ridged surface and was mixed with 10 μL of thrombin solution, giving a final fibrinogen concentration of 0.3 mg/mL and final thrombin concentration of 1 U/mL on the slide. The samples were sealed inside a petri dish along with damp paper to ensure a moist atmosphere for the sample and left to polymerize for one hour at 37°C [14].

After polymerization, a pipette was used to gently remove a superficial fibrin mesh, leaving a 2-D fibrin network on the ridged surface. In experiments where beads were used, 30 μL of a 20 nm carboxy-coated microsphere (FluoSpheres^™^ Carboxylate-Modified Microspheres, Invitrogen by Thermo Fisher Scientific/Life Technologies Corporation, Eugene, OR) (interchangeably referred to as beads) solution, at the specified dilution, was sonicated for 5 minutes and added to the sample. The bead solution was incubated with the fibrin for 1 minute at room temperature and then gently pipetted off. The sample was then washed three times with 30 μL of HBS buffer to ensure removal of any excess bead solution. Following the washes, 20 μL of HBS buffer was placed on top of the sample prior to conducting microscopy imaging.

### Microscopy

**Fig 1:**
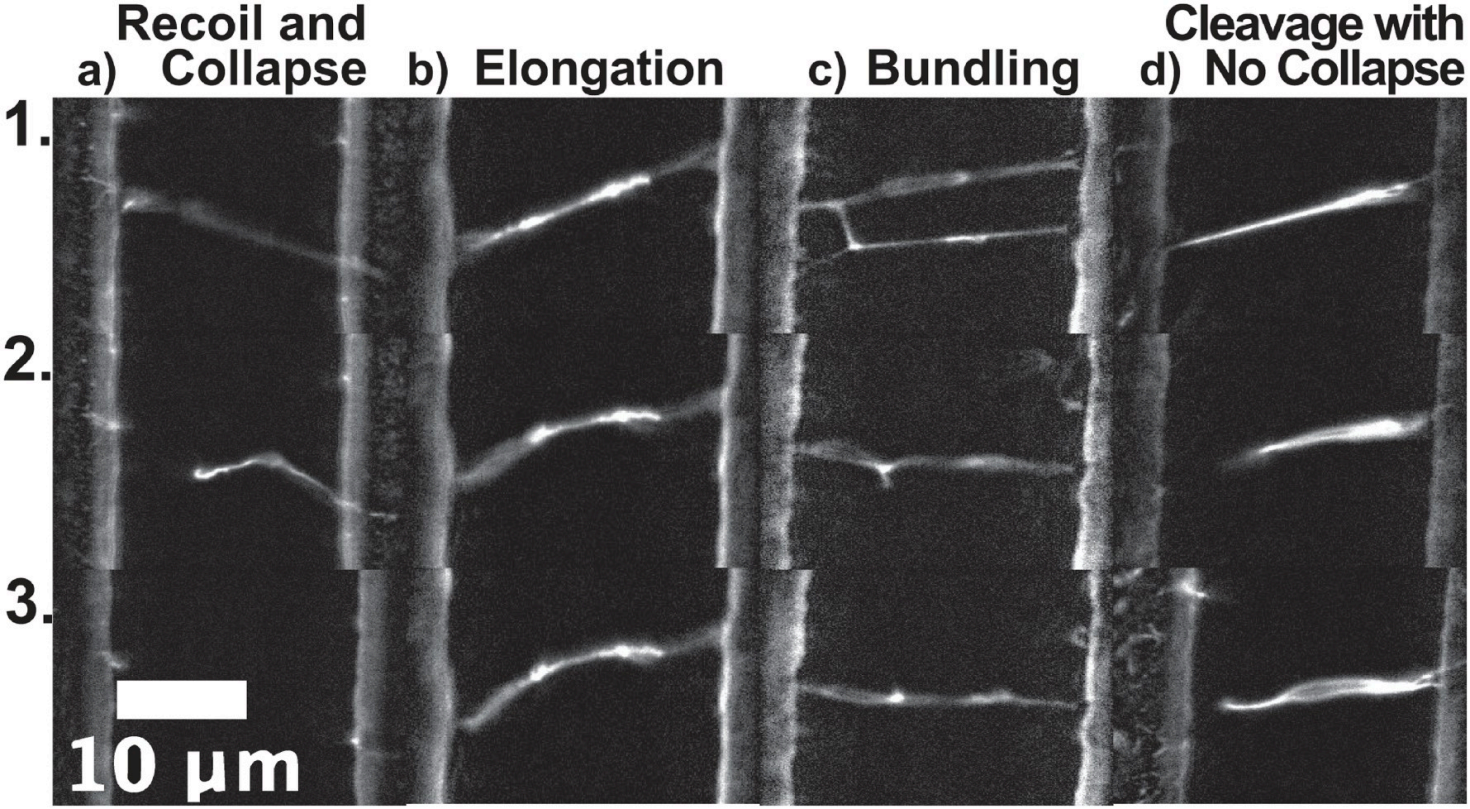
Representative Time Series of Observed Fibrinolytic Outcomes: Each image on top (panel 1) displays the fiber’s initial conformation. Time increases as the image series progresses downwards. Final frames are shown in panel 3. Distinct image series show a) fibers are cleaved, recoil, and collapse (See S1 Movie); b) fibers remain uncleaved and elongate (See S2 Movie); c) one fiber is cleaved and bundles with another intact fiber (See S3 Movie); and d) fibers are cleaved but do not collapse (See S4 Movie).

Samples were imaged on a Lieca DMi8 epifluorescent microscope (Leica Microsystems Inc., Buffalo Grove, IL), using a 63x oil-immersion objective. Using light with a wavelength of 480 nm, corresponding to the excitation wavelength of ALEXA-488 labeled fibrinogen, an imaging area was located. Sample areas containing at least 4 individual fibers, each separated by at least 1μm within the camera frame were selected for imaging to maximize the rate of data acquisition. Once an area was found, the microscope settings were changed to a wavelength of 553 nm, the excitation wavelength of the carboxylate-coated polystyrene beads (580 nm) under investigation. We located the fibers using the 480 nm wavelength to minimize fluorescent excitation of the beads while locating the fibers, because the bead excitation spectrum has a minimum ~480 nm, per the manufacturer’s data sheet. This was done because we suspected that the beads may be sensitive to light dosage. While later experiments did not bear this hypothesis out (see results), we maintained this experimental approach. To initiate lysis, plasmin (Enzyme Research Laboratories, South Bend, IN) was added directly to the sample and then time-series acquisition began immediately afterwards. After the addition of plasmin, the attached microscope camera (Leica DFC9000GT SCMOS 4 Megapixel monochrome camera) captured an image every 30 seconds for one hour. The camera shutter was set to remain open during the entire imaging process, unless otherwise stated. An example time series is displayed in Fig 1.

### Image analysis

When the time-lapse was completed, the file was imported into FIJI image analysis software where the singular fibers were analyzed [19]. In the initial frame, single fibers, all of which presented as tensed structures, were counted and designated for further analysis. Cleaving times were found by analyzing each image series frame-by-frame until finding the frame in which the fiber cleaved. The number of frames prior to cleavage was multiplied by the time interval between frames to find the cleavage time. Additionally, during these intermediate frames the occurrences of bundling were quantified, categorized as two neighboring, largely disconnected fibers, joined to form a singular new fiber (See S3 Movie). Over the course of imaging, many fibers lost the tension they presented in the initial frames and became loose structures. We identified these fibers that lost their initial tension as “elongated” (See S2 Movie), and they were counted in the final frame of the timelapse, along with any fibers that remained tensed.

### Bead concentration experiments

The beads were provided as 2% solids, and a calculation of the bead concentration was done as follows (per the manufacturer):

$$\frac{Number\;of\;Beads}{mL}=\frac{6C\times 10^{12}}{\rho \times \pi \times d^{3}}$$

where *C* is the concentration of suspended beads in weight volume % per 100g of beads in 1 mL (0.02 for 2% solids), *ρ* is the density of bead material (1.05 g/mL for polystyrene), and d is the diameter of microspheres in μm. The beads used here are 0.02 μm in diameter, yielding a stock bead concentration of 4.55x10^12^ beads/μL, which corresponds to roughly 714 beads/fiber volume (.16 μm^3^) in solution, assuming a fiber length and diameter of 20.0 μm and 100 nm, respectively [20]. The number of beads bound to the fiber depends on this bead concentration as well as the binding kinetics (on- and off-rates) of the bead-fibrin interaction, which are unknown, and the length of time that the beads are incubated with the fiber network (~ 1 minute).

**Fig 2:**
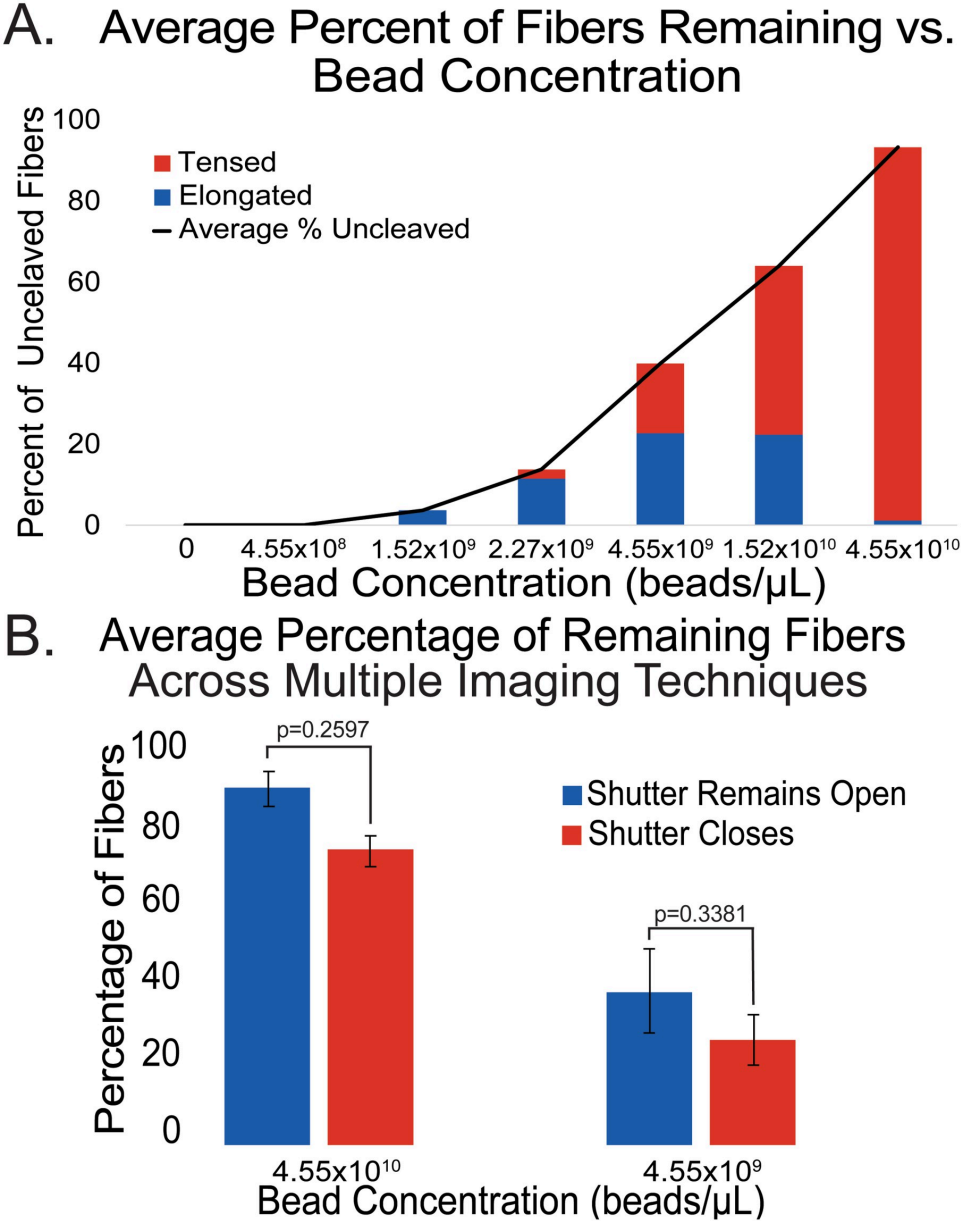
Percentage of Fibers Remaining After One-Hour of Imaging at Various Bead Dilutions and Various Light Doses: (A) The height of the bars represents the percentage of the total number of fibers that remain uncleaved after 1 hour of plasmin digestion. The bars are subcategorized by color to represent the fraction of uncleaved fibers that either remain tense (red) or elongate (blue). The total number of fibers analyzed for each of the bead concentrations are as follows: Nfiber = 32 (4.55x10^8^ beads/μL); 85 (1.52x10^9^ beads/μL); 82 (2.27x10^9^ beads/μL); 103 (4.55x10^9^ beads/μL); 86 (1.52x10^10^ beads/μL); 85 (4.55x10^10^ beads/μL). Note that fiber bundling is not differentiated in this plot but is in Fig 4. (B) The height of the bars represents the percentage of uncleaved fibers present after an hour of imaging. Data from two bead concentrations are presented. The bars are categorized by color based on the amount of light presented to the sample over the course of imaging; either constant light conditions with the camera shutter remaining open (blue) or low light conditions with the camera shutter closing after each frame of the time-lapse (red). At both 4.55x10^10^ beads/μL and 4.55x10^9^ beads/μL bead concentrations, there is no statistical difference between the percentage of uncleaved fibers with p-values of 0.2597 and 0.3381, respectively. The total number of fibers analyzed at either concentration in constant light conditions (blue bars): Nfiber = 85 (4.55x10^10^ beads/μL), 103 (4.55x10^9^ beads/μL). The total number of fibers analyzed at either concentration in low light conditions (red bars): Nfiber = 49 (4.55x10^10^ beads/μL), 93 (4.55x10^9^ beads/μL). The error bars represent of standard error.

To test the effect of bead concentration during labeling on fibrinolytic results, a dilution series of stock bead solutions was made with HBS buffer. Dilutions from stock of 1:100 (4.55x10^10^ beads/μL), 1:300 (1.52x10^10^ beads/μL), 1:1000 (4.55x10^9^ beads/μL), 1:2000 (2.27x10^9^ beads/μL), 1:3000 (1.52x10^9^ beads/μL), and 1:10,000 (4.55x10^8^ beads/μL) were tested, corresponding to bead concentrations of: 7.1, 2.3, 0.7, 0.36, 0.24, and 0.07 beads/fiber volume, respectively. Dilution factors were chosen based on the results shown in Fig 2, which suggest little difference in results outside of this dilution range. Five independent clot samples were made as previously described and then labeled with beads at one of the concentrations listed, as to have five trials at each concentration. After labeling the samples with fluorescent beads, the samples were taken to the microscope for imaging. Plasmin was added to these samples to reach final concentration of 1.0 U/mL and digestion images were collected every 30 s.

### Plasmin concentration experiments

To test the effect of plasmin concentration on the lysis of bead-labeled fibers, trials at varying plasmin concentrations were conducted. Samples were made as described previously and labeled with a bead solution at 4.55x10^9^ beads/μL concentration. After labeling, samples were visually searched using 480 nm light (corresponding to the ALEXA-488 wavelength) to find areas with individual fibers while minimally exciting the beads. The wavelength was changed to 553 nm (corresponding to the fluorescent bead excitation wavelength (580 nm)), plasmin was added, and images were collected every 30 s for one hour. Plasmin (Enzyme Research Labs, 11.03 U/mL, stock concentration) was aliquoted and frozen (-20° C) at concentrations that were double the desired final concentration. During an experiment, one aliquot was thawed and added to the fibrin-network containing solution at equal volumes (final total volume of 20 μL), thereby halving the plasmin concentration. Five trials were conducted at each of the following final plasmin concentration, 0.7 U/mL, 1.0 U/mL, and 2.0 U/mL.

### Computer simulations

To test mechanisms suggested by experiments, computer simulations of 2-dimensional bead diffusion and binding to fibrin were conducted. Simulations were run with MATLAB R2016a software using custom code. The simulation domain was a 2 μm by 2 μm box with reflecting boundary conditions on the top, left, and right, and with an absorbing boundary condition on the bottom. The bottom of the domain represented a fibrin fiber, and hence the absorbing boundary condition modeled irreversible binding of the beads to the fiber. The domain was partitioned into a grid using space steps of 0.02 μm in both the x- and y-directions. In a single simulation, 100 beads (20 nm diameter) were uniformly and randomly distributed on grid nodes in the top half of the simulation domain. At each time step, each bead could remain at its current grid node or move to one of the 4 nearest neighbor grid nodes, with equal probability. The time step, 1x10^-5^ s, was approximated from the diffusion coefficient of a 24 nm diameter bead (D≈1x10^-7^ cm^2^/s) and the 0.02 μm space step via: $D\approx \frac{{\left(0.02\;\mu m\right)}^{2}}{4\times time\;step}$ [21]. Each simulation was run for 60 seconds of simulation time. 500 independent simulations were run, and data was collected about if/when and where each bead bound to the fiber (See S5 Movie).

### Statistical analysis

Much of the data presented in the paper consists of counts of events or the percentage of one type of event occurring out of the total events. When testing differences between two results (counts or percentages), the statistical significance was determined by a two-tailed ANOVA test with a p-value cutoff of 0.05.

When investigating the relationship between percentage measurements and concentration of beads or enzymes, regression analyses were performed in Rstudio [22]. Percentages of fibers from each independent experiment were plotted as a function of experimental condition (bead or enzyme concentration), and linear regression was used to determine whether the concentration dependence was significant.

Throughout the text and displayed in figures, error values or bars are representative of standard error.

## Results

By analyzing the lysis of small fibrin networks labeled with fluorescent beads, we determined that multiple lytic outcomes are possible: (1) normal cleavage along the length of the fiber and subsequent digestion (See S1 Movie), (2) cleavage of fibers at the ridge connection and then no further digestion observed (See S4 Movie), (3) no cleavage event and instead fiber elongation over the course of imaging (See S2 Movie), and (4) cleavage of one or more fibers that bundle together to form a new fiber (See S3 Movie). Fig 1 shows an exemplary time series for each of these outcomes and S1–S4 Movies show time series of the different events. Outcomes such as cleavage and bundling have been observed previously in experiments without beads [8, 10], so are likely not solely a result of their utilization. We then sought to test how these outcomes were affected if different bead concentrations were utilized.

### Bead concentration and percentage of fibers uncleaved and/or elongated

The bead concentrations tested were, in order of increasing concentration, 4.55x10^8^, 1.52x10^9^, 2.27x10^9^, 4.55x10^9^, 1.52x10^10^, and 4.55x10^10^ beads/μL, using a fixed plasmin concentration of 1 U/mL. Results from these tests showed a strong correlation between the percentage of fibers cleaved and bead concentration (Fig 2). At the lowest concentration tested, 4.55x10^8^/μL, all observed fibers were cleaved, and at the next lowest concentration, 1.52x10^9^ beads/μL, 3.6 ± 3.6% of fibers remained after an hour of continuous imaging during digestion. Further, 13.7 ± 6.2% remain at 2.27x10^9^ beads/ μL, 39.8 ± 10.7% remain at 4.55x10^9^ beads/μL, 63.9 ± 1.3% remain at 1.52x10^10^ beads/μL, and 93.2 ± 4.4% remain at 4.55x10^10^ beads/μL. Alongside this cleavage trend, the fibers that were not cleaved also showed the tendency to elongate at lower bead concentrations. At the highest tested bead concentration, 4.55x10^10^ beads/μL, only one fiber elongated (out of 81), corresponding to only 1.2% of fibers remaining at this dilution. All non-elongated, non-cleaved fibers maintained their inherent tension present at the beginning of data collection. As bead concentration was decreased, the rate of elongation rose; 34.9% of remaining fibers elongated at 1.52x10^10^ beads/μL, 56.8% at 4.55x10^9^ beads/μL, 83.3% at 2.27x10^9^ beads/μL, and 100% at 1.52x10^9^ beads/μL, but it is important to note that the number of uncleaved fibers also decreased, so there were fewer fibers that could have elongated. Both trends are displayed graphically in Fig 2A.

### Effects of light dosage on results

To test whether light dosage affected our results we performed experiments where the shutter remained open during the entire plasmin degradation process and compared the results with those from experiments where the shutter was only open during image acquisition. Images were collected every 30s with an exposure time of 200 ms. We measured the light power at the surface of the glass using a power meter (Ophir StarLite 7Z01565). Under identical conditions to those used during the experiments, 160–180 μW of power was measured. Thus, for experiments where the shutter was left open during the one-hour image acquisition period, 0.612 J of energy would be deposited into the sample, while in experiments where the light was only on during image acquisition, 0.0041 J of energy would have been deposited, 150-fold less energy. Data was collected for two bead concentrations, 4.55x10^10^ beads/μL and 4.55x10^9^ beads/μL at a plasmin concentration of 1.0 U/mL. Results are shown in Fig 2B. While the mean percentage of uncleaved fibers was higher for fibers that were continuously exposed to light, a two-sample t-test analysis did not indicate that the difference was statistically significant. At a concentration of 4.55x10^10^ beads/μL, 134 fibers were analyzed; 85/134 were imaged with the camera shutter constantly open and 49/134 were imaged with the camera shutter only open during image acquisition. At a concentration of 4.55x10^9^ beads/μL, 196 total fibers were analyzed; 103/196 were imaged with the camera shutter constantly open and 93/196 were imaged with the camera shutter only open during image acquisition.

### Effects of plasmin concentration on cleavage and elongation

**Fig 3:**
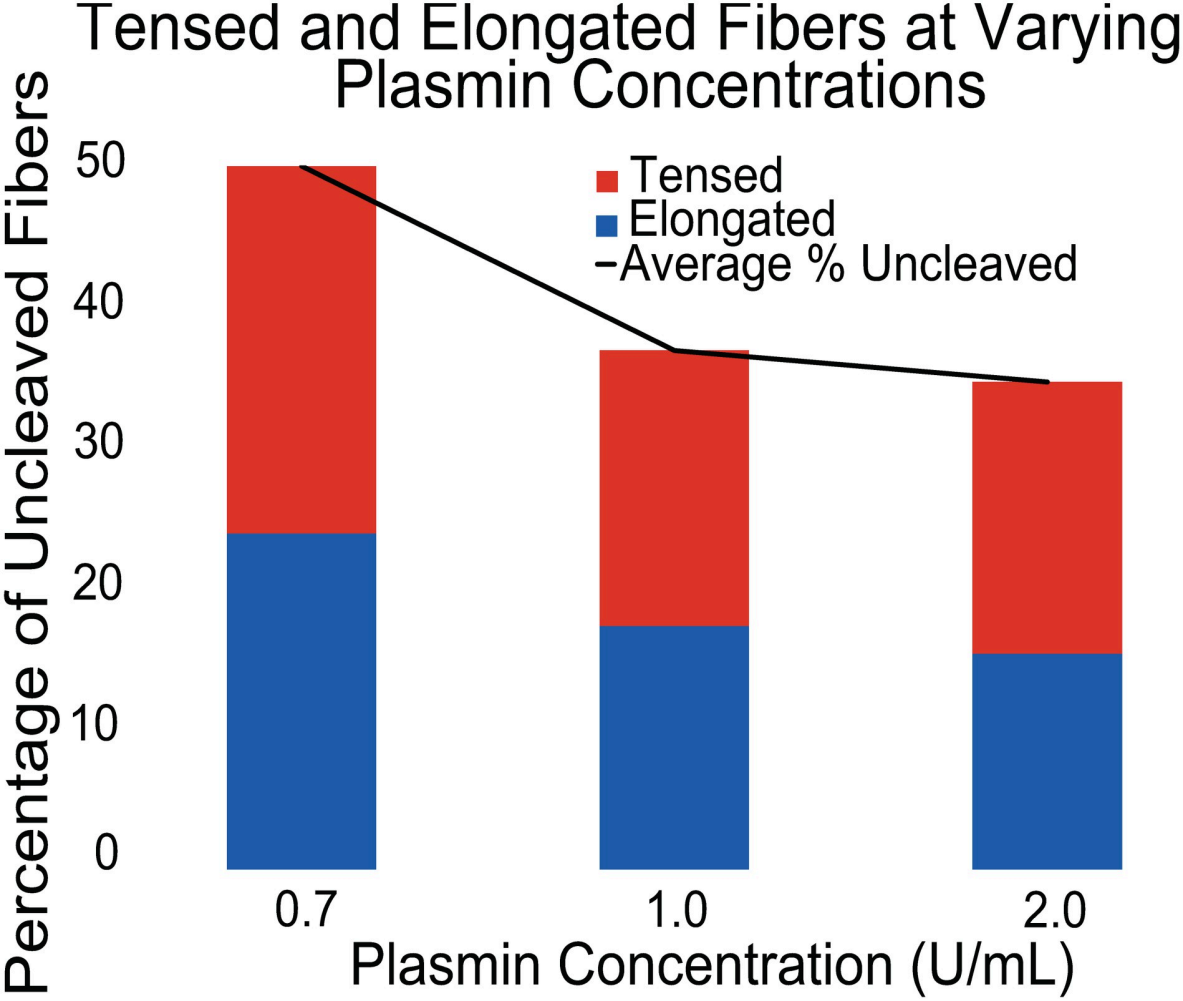
Percentages of tensed and elongated fibers at various plasmin concentrations: The height of the bars displays the average percentage of uncleaved fibers at each tested plasmin concentration after 1 hour. The bars are further divided into two categories denoted by color; the percentage of uncleaved fibers that remained tensed (red) or elongated (blue) throughout the course of imaging. All data was collected using fibrin fibers labeled with a bead concentration of 4.55x10^9^ beads/μL.

Using a fixed 4.55x10^9^ beads/μL concentration, we varied the plasmin concentration to test its effect on these results. At the lowest plasmin concentration tested, 0.7 U/mL, 48.5 ± 19.5% of fibers initially present remained through the duration of data collection. At a plasmin concentration of 1.0 U/mL, 35.6 ± 21.8% of fibers remained (agreeing with our previous results), and at 2.0 U/mL plasmin, 34.6 ± 12.7% of initial fibers last through data collection. Elongation of persisting fibers throughout these trials at differing plasmin concentration remained roughly constant. At a plasmin concentration of 0.7 U/mL, 47.8% of fibers elongate, compared to 46.9% and 44.2% of fibers displaying elongation at 1.0 U/mL and 2.0 U/mL plasmin, respectively. This falling percentage of fibers remaining, and constant rate of elongation are shown in Fig 3.

### Effects of fiber bundling on elongation

**Fig 4:**
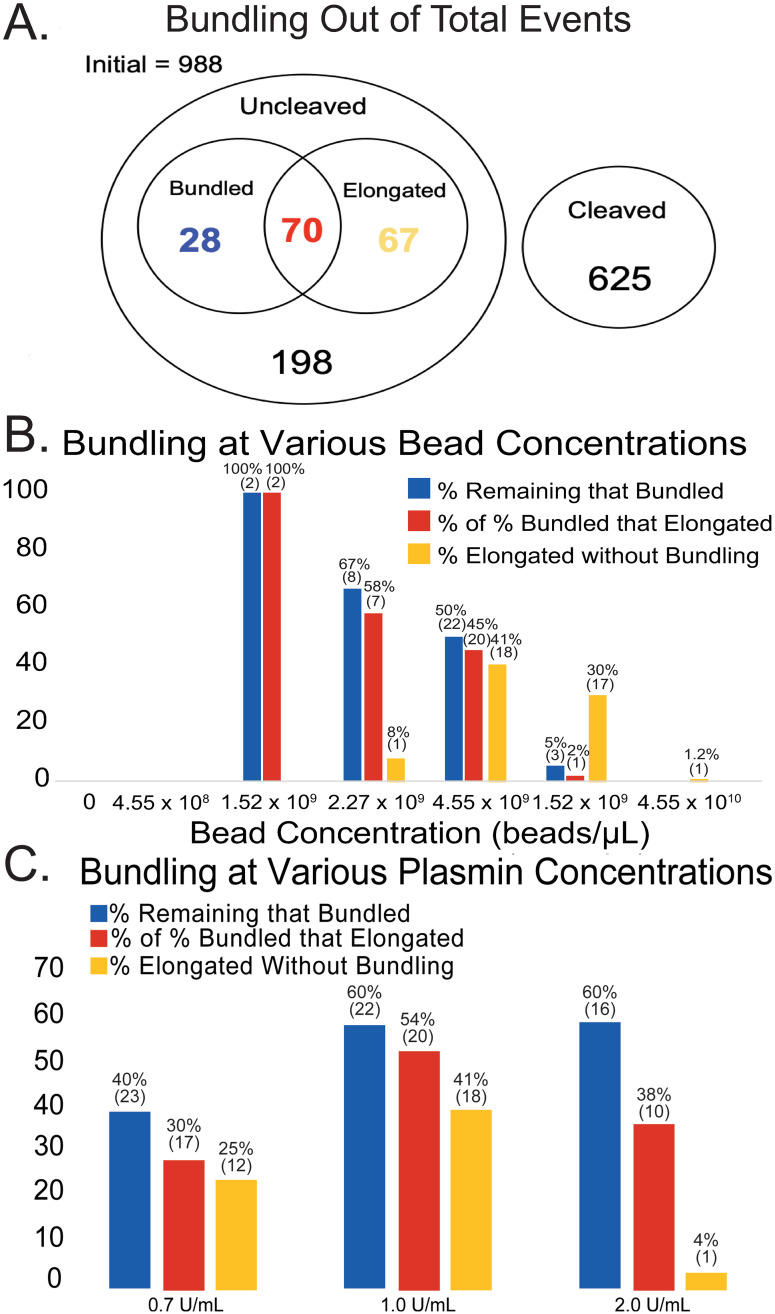
Bundling events. (A) A Venn Diagram displaying how many fibers were cleaved and uncleaved and of the uncleaved fibers, how many were bundled and straight (blue), bundled and elongated (red) and elongated (yellow). (B) Plot displaying bundling as a function of bead concentration. Blue bars denote the percentage of uncleaved (remaining) fibers that bundled, red bars show the percentage of uncleaved fibers that bundled and elongated, and yellow bars represent the percentage of uncleaved fibers that elongated without bundling. (C) Bar plot displaying bundling data broken down by plasmin concentration. Note that in all panels, fibers comprising the red bars are a subset of the fibers comprising the blue bars.

Often, in experiments, fiber fragments from a cleaved fiber would bundle with an uncleaved fiber, creating a thicker fiber. Sometimes the ends of two cleaved fibers would stick together and create a new fiber. For counting purposes, we count each of these situations as one bundled fiber because both situations result in a single fiber. Described in Fig 4, bundling of fibers was very prevalent in this study across all plasmin concentrations and bead dilutions. Previous experiments looking at fiber bundling [8, 14] looked at either 2-D or 3-D networks of relatively densely packed fibers. In our experiments, we mostly consider individual fibers that are spatially separated from another fiber by at least 1.0 μm. Thus, the initial probability of bundling in our experiments is much lower than it would be for higher density networks. Despite this, fiber bundling was a commonly observed result.

Taken across all experiments, out of the 988 fibers observed 363 were not cleaved by plasmin during data collection. Of these 363 fibers, 27.0% bundled with another fiber during this process. Typically, fiber bundling occurred when the remnants of a cleaved fiber bound to a neighboring, uncleaved fiber. The majority, 71.4%, of bundled fibers also elongate. This percentage is in stark contrast to the 18.5% (67/363) of fibers that show elongation without undergoing a bundling event first. Shown in Fig 4B, at a 4.55x10^10^ beads/μL concentration, 81/85 fibers persisted throughout plasmin exposure, but only one fiber showed elongation, and it did so without bundling. Decreasing the bead concentration from 4.55x10^10^ beads/μL to 1.52x10^10^ beads/μL shows more fibers elongating without bundling: 55/86 fibers persisted, and 17/55 remaining fibers elongated without bundling. Only 3/55 remaining fibers bundled, and of those three, one elongated and two retained their tension. At a concentration of 4.55x10^9^ beads/μL 44/103 fibers persisted. Of those 44 fibers, 20 fibers bundled and then elongated, while 18 fibers elongated without bundling, 2 fibers bundled but didn’t elongate, and 4 fibers remained tense. Thus, 90.9% of fibers that bundle at this concentration show elongation. A 2.27x10^9^ beads/μL concentration showed an increased number of cleaved fibers, with only 12/82 fibers persisting. Of these remaining fibers, 8 fibers bundled, 7 of which elongated (87.5%), only one fiber elongated without bundling, and 3 fibers remained tensed. At 1.52x10^9^ beads/μL, only 2/87 fibers persisted, and both of those fibers bundled with other fiber fragments and elongated. A linear regression analysis of these results indicated that elongation without bundling is independent of bead concentration, but that bundling and elongation with bundling do depend on bead concentration (Fig S1 in S1 File).

Fig 4C displays similar data, but as a function of plasmin concentration, rather than bead concentration (which was held constant at 4.55x10^9^ beads/μL). At a plasmin concentration of 0.7 U/mL, 48/96 fibers persisted, with 23/48 observed fibers bundling, 17/23 of these elongated (73.9%), and 12/48 fibers elongated without bundling. With 1.0 U/mL plasmin, 47/118 fibers remained, with 24/47 fibers bundling, 21/24 of which elongated (87.5%), and 18/47 elongated without bundling. Finally, at a plasmin concentration of 2.0 U/mL, 25/75 fibers persisted, with 16/25 fibers remaining because of bundling, 10/16 later elongated (62.5%), and 1/25 fibers elongated without a bundling event. A linear regression analysis of these results indicated that elongation without bundling is dependent on the plasmin concentration, but that bundling and elongation with bundling are independent of plasmin concentration (Fig S2 in S1 File).

As a comparison, we also analyzed fibers that were not labeled with beads at all. In this case, imaging was performed utilizing a wavelength of 480 nm, corresponding to the ALEXA-488 excitation wavelength. In looking at 455 fibers with plasmin added at a final concentration of 0.066 U/mL, only 3% (14/455) of fibers bundled, and all of these occurred in areas of higher local fibrin fiber density. Importantly, all these fibers, labeled with only ALEXA-488, recoiled backwards or were digested away rather than persisting throughout image acquisition like observed in some bead labeled fibers.

### Location of the cleavage site along the fiber as a function of bead concentration

**Fig 5:**
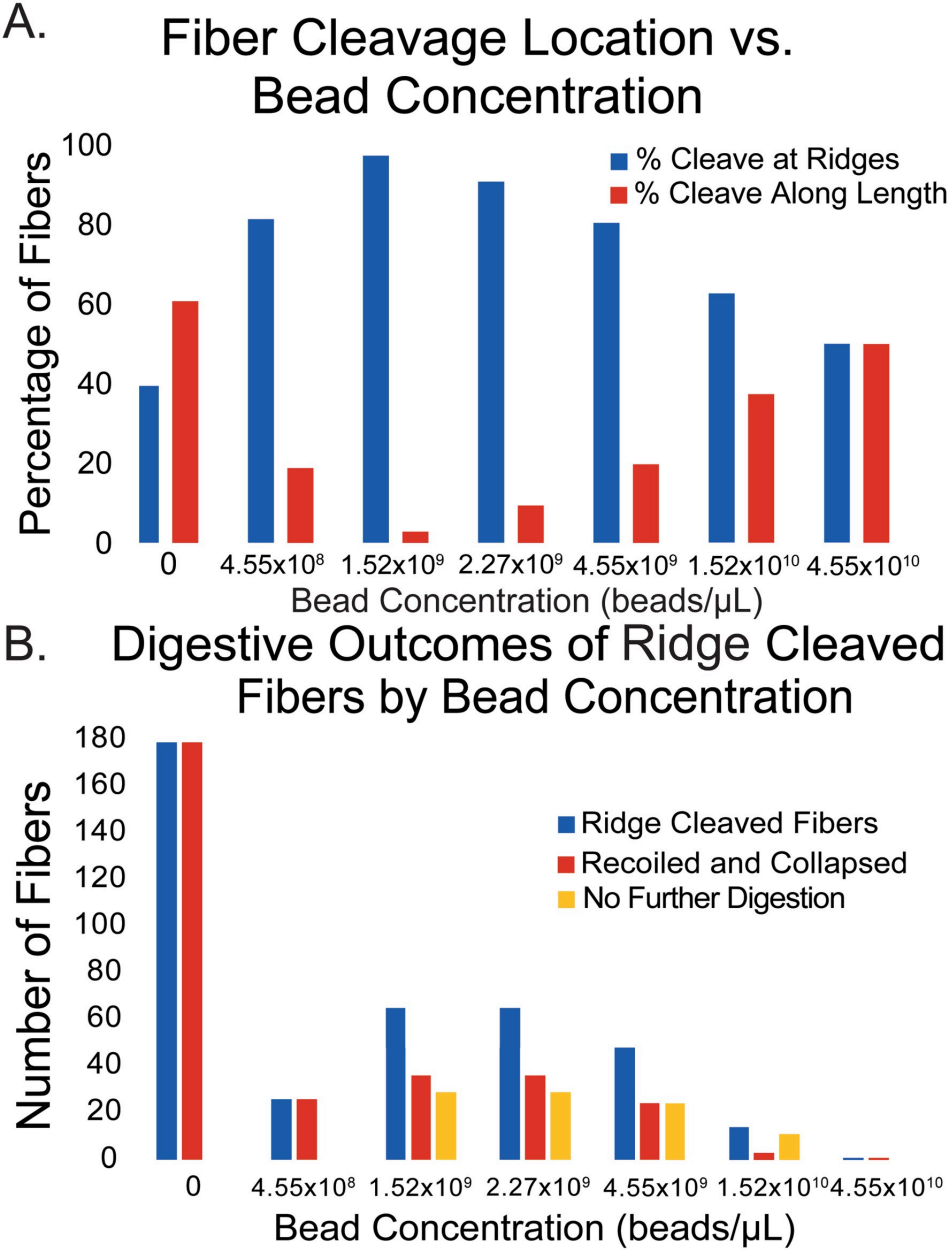
Cleavage location and digestive outcomes of ridge cleaved fibers. (A) The percentage of fibers that cleaved at either the fiber-ridge connection or elsewhere along the length of the fiber are presented for each bead concentration. Percentages of fibers that cleaved at the fiber-ridge connection are shown in blue, and fibers that cleaved somewhere along the length of the fiber are shown in red. (B) Of the ridge cleaving fibers, the digestive outcomes are further broken down by bead concentration. The blue bars are representative of the total number of ridge cleaving fibers at each dilution. Of this total (1.52x10^9^ beads/μL = 65, 2.27x10^9^ beads/μL = 65, 4.55x10^9^ = 48, 1.52x10^10^ beads/μL = 14, 4.55x10^10^ beads/μL = 1), fibers either recoiled and collapsed (red) or showed no signs of further digestion (yellow).

Next, we investigated the location of cleavage events and found that bead concentration influences the site of cleavage along a fiber. At a bead concentration of, 1.52x10^9^ beads/μL, 97.2% of fibers had their cleavage event occur at the ridge of the optical adhesive substrate, while the remaining 2.8% cleaved at some non-specific point along the length of the fiber. Increasing the bead concentration to 2.27x10^9^ beads/μL resulted in 90.7% of fibers cleaving at the ridge-fiber connection and 9.3% cleaving along the length of the fiber. Increasing the bead concentration again to 4.55x10^9^ beads/μL continued this trend with 80.3% cleaving at the ridge and 19.7% cleaving along the length of a fiber. At a bead concentration of 1.52x10^10^ beads/μL, 62.6% of fibers cleaved at the ridge and 37.4% cleaved along the length of the fiber. Finally, at the highest tested bead concentration, 4.55x10^10^ beads/μL, 50.0% of fibers cleaved at the ridge and 50.0% of fibers cleaved along the length of a fiber. This correlation is shown in Fig 5A. As a comparison, we also looked at the cleavage site of fibers that had no beads. 39% (179/455) cleaved at the ridge, while 61% (276/455) cleaved somewhere along the fiber length.

Fibers that cleaved at their connection to the ridge then showed two digestive outcomes: (1) they collapsed back to the connected ridge as expected, or (2) they persisted throughout the remainder of data collection connected to one ridge and did not display signs of further collapsing. Across all fibers observed that cleaved at the ridge connection, 224 fibers displayed the expected recoil and digestion after cleaving. 248 fibers in the study were observed cleaving at a ridge connection and then showed no further signs of digestion. These digestion outcomes are shown in Fig 5B. A linear regression analysis of these results indicated that the percentage of fibers experiencing no further digestion was independent of bead concentration, while the percent of fibers that recoiled and collapsed significantly depend on bead concentration (Fig S3 in S1 File).

### Cleavage time as a function of bead concentration

**Fig 6:**
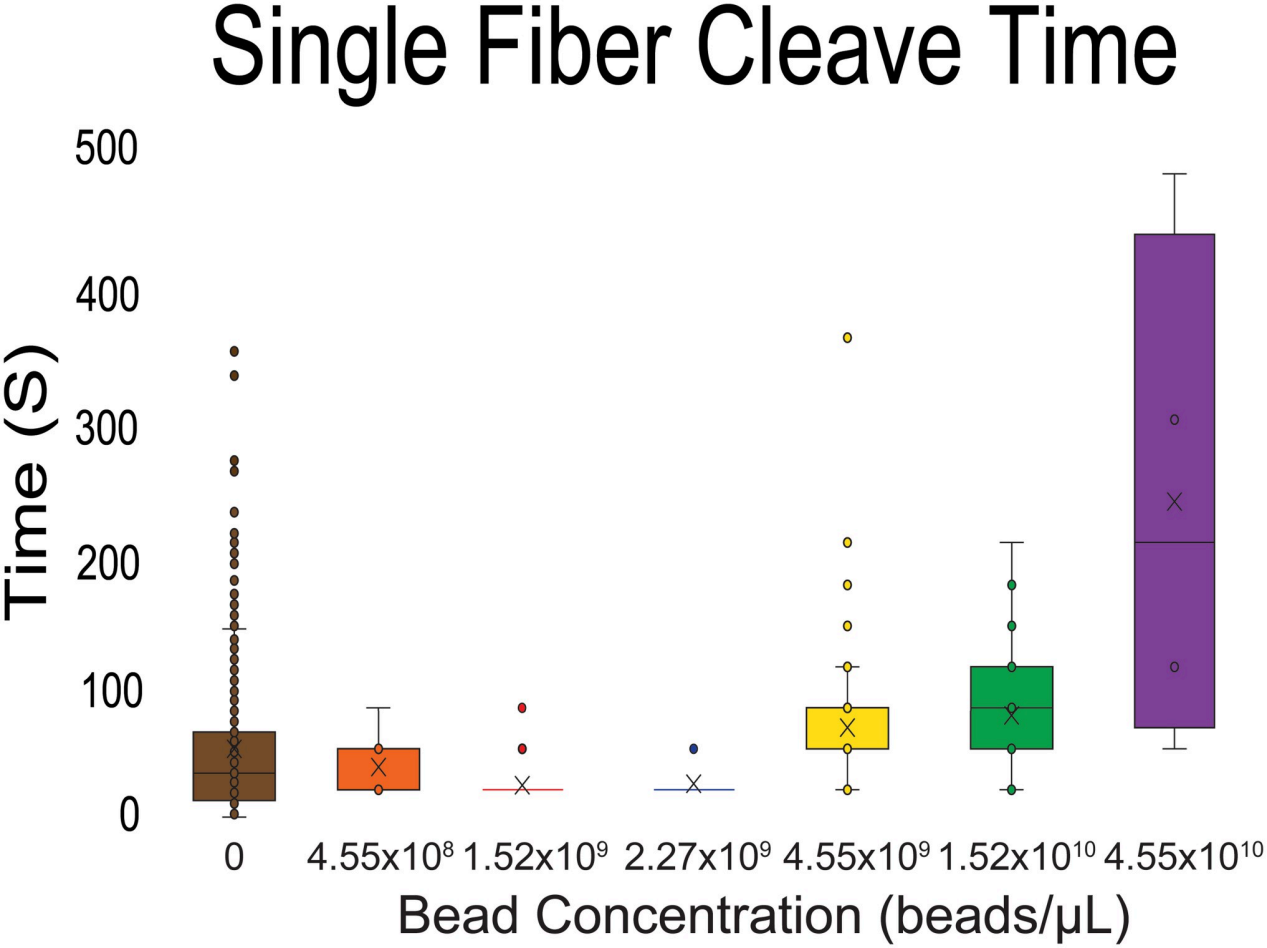
Box and whisker plots of cleavage time as a function of bead concentration: Cleavage time was calculated as the time between the addition of plasmin and the first image in the time series where cleavage was observed. This frame number was multiplied by the frame interval (30 s) and 30 s were subtracted to adjust for first image in the time-lapse (captured at the time of plasmin addition). The number of fibers used to calculate the mean cleavage time at each concentration is as follows: Nfiber = 5 (4.55x10^10^ beads/μL), 31 (1.52x10^10^ beads/μL), 49 (4.55x10^9^ beads/μL), 70 (2.27x10^9^ beads/μL), and 85 (1.52x10^9^ beads/μL). The calculated mean cleavage times (represented as an X on the box and whisker plot) are as follows: 4.55x10^10^ beads/μL = 240 ± 85 s, 1.52x10^10^ beads/μL = 84 ± 20 s, 4.55x10^9^ beads/μL = 75 ± 23 s, 2.27x10^9^ beads/μL = 35 ± 5 s, and 1.52x10^9^ beads/μL = 34 ± 5 s. The “box” represents the first and third quartile (the interquartile range) of fiber cleavage times for each data set, and where applicable, the horizontal line inside the box signifies the median of the data set. The “whiskers” represent values 1.5x above and below the interquartile range of cleavage times of each data set and are as follows: 4.55x10^9^ beads/μL = 30 s and 120 s, 1.52x10^10^ beads/μL = 30 s and 210 s, and 4.55x10^10^ beads/μL = 60 s and 480 s. Where applicable, cleavage times falling outside the whiskers are represented as dots.

Bead concentration is also a determinant of the length of time it takes for a fiber to cleave. Fig 6 visually describes this phenomenon, where high bead concentrations induced the longest average cleaving time of fibers. At the highest tested concentration, 4.55x10^10^ beads/μL, the mean cleavage time was 240.0 ± 84.9 seconds after the addition of plasmin. At a concentration of 1.52x10^10^ beads/μL, this timeframe was reduced to 84.19 ± 20.0 s. Lowering the bead concentration from 4.55x10^9^ beads/μL, 2.27x10^9^ beads/μL, and 1.52x10^9^ beads/μL, the mean cleavage times dropped to 75.31 ± 22.9 s, 34.72 ± 4.9 s, and 33.53 ± 4.8 s, respectively.

### Computer simulation results of bead diffusion and binding

**Fig 7:**
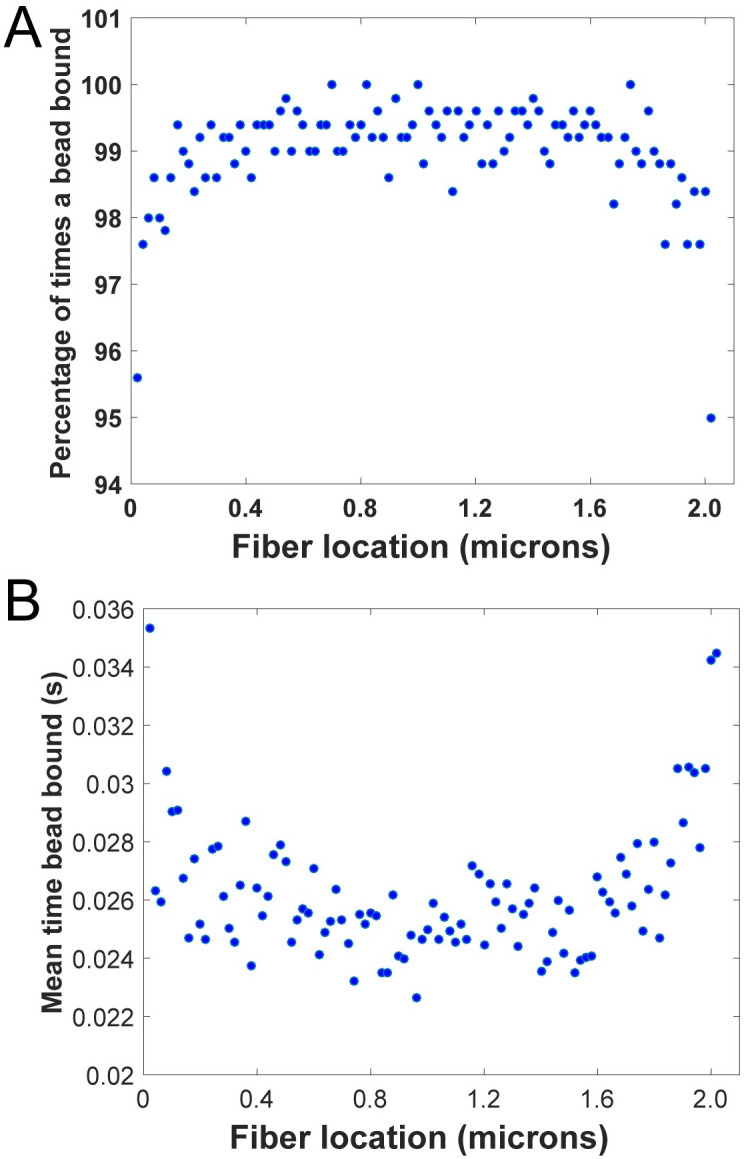
Simulation Results of Bead Diffusion and Binding: (A) The percentage of times a bead bound (out of 500 independent simulations) as a function of location along the fiber. Locations 0 and 2.0 are where the fiber connects to the ridges. (B) The mean time it took a bead to bind to the fiber (averaged over 500 independent simulations) as a function of location along the fiber. Locations 0 and 2.0 are where the fiber connects to the ridges.

Computer simulations reveal how the location along a fiber affects the frequency with which a bead binds (Fig 7A) and the length of time it takes a bead to bind (Fig 7B). Beads are less likely to bind to the locations where the fiber connects to the ridge (locations 0 and 2.0 μm, Fig 7A), and when beads do bind to those locations, it takes them longer, on average, than it does for beads to bind to other locations along the fiber (Fig 7B). S5 Movie shows a representative time series of one of the diffusion simulations.

## Discussion

Fibrinolysis assays have the potential to both reveal biophysical and biochemical processes that play a role in blood clot digestion and help to diagnose pathologies. In developing such assays meant to mimic *in vivo* systems, it is vital to ensure that molecular markers added to the system do not alter the physiological processes in some way. Because of the observed fiber elongation in previously published results regarding fibrinolysis [12, 13], we have here undertaken a systematic study of the effects of nanoscopic beads, used to label fibrin fibers, on fibrinolytic processes. Our results are in qualitative agreement with another recent report that also addressed the effects of bead labeling on fibrinolysis [15], but important quantitative differences exist and will be addressed in the discussion. Moreover, we investigated fiber bundling, the diffusion of beads onto fibers, and cleavage locations and rates, which were variables not considered in other studies.

Our results indicate that the carboxy-coated nanospheres (beads) can affect fibrinolytic outcomes via several distinct mechanisms. As shown in Fig 2, at high bead concentrations, such as 4.55x10^10^ beads/μL, there were nearly no cleavage events observed. As the fluorescent bead concentration decreased, there was an increase in fiber cleavage events and a decrease in the percentage of fibers that remained throughout the course of imaging. These trends are in qualitative agreement with a recent report by Rimi and Helms [15], but results at specific concentrations often differed. For example, at 4.55x10^9^ beads/μL, Rimi and Helms found that <10% of fibers were cleaved, while in our experiments > 60% of fibers were cleaved [15]. These differences could be due to the slightly different plasmin concentrations (0.75 U/mL vs 1 U/mL), thrombin concentrations (0.1 U/mL vs 1.0 U/mL) or FXIIIa crosslinking. In any case, the trend clearly shows a strong dependence of a fiber’s lytic susceptibility to the concentration of beads used to label it.

**Fig 8:**
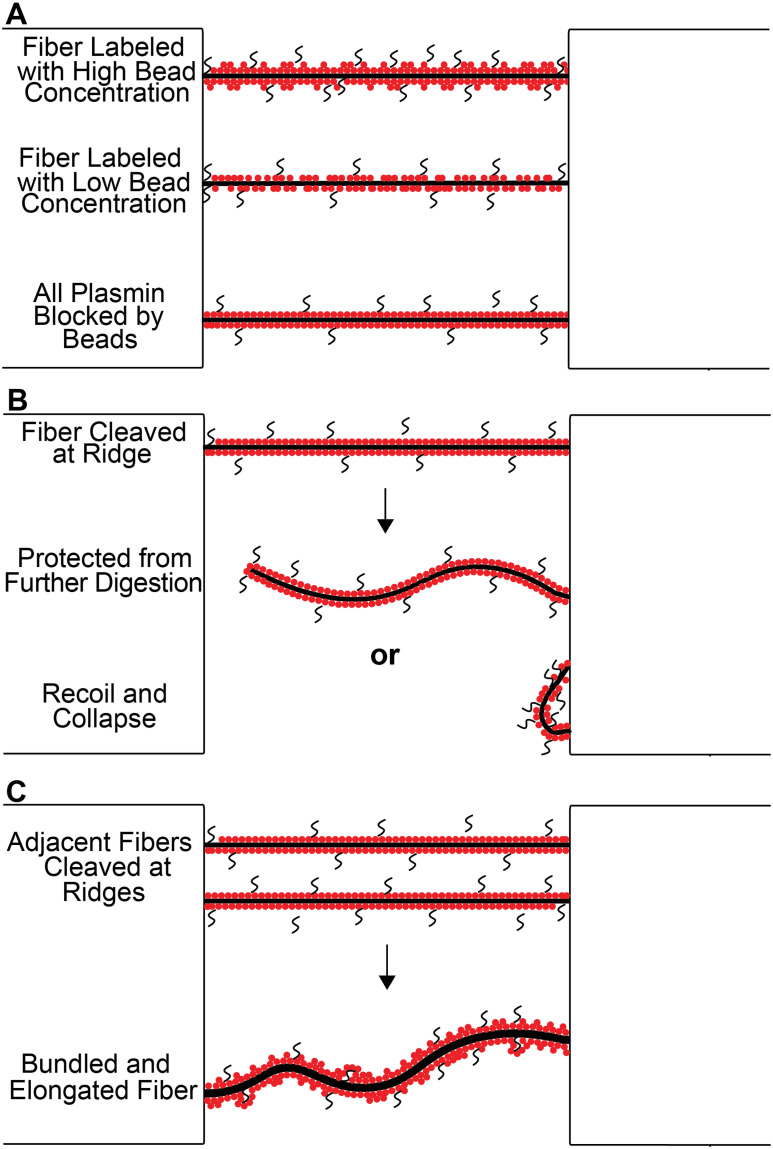
Cartoon Model: (A) The proposed sheathing mechanism and the disparity between cleavage location at differing bead dilutions are shown for three representative fibers, denoted as black lines. Fluorescent beads are shown as red circles and plasmin is represented as a black s-shape. The uppermost fiber is indicative of plasmin binding locations being dispersed evenly across a fiber when high fluorescent bead concentrations are utilized. The middle fiber represents the preferential ridge cleavage of fibers labeled with lower bead concentration, as the beads fill the length of the fiber, blocking most plasmin binding sites rather than the ridge locations. The bottom fiber represents a scenario where a fiber remains uncleaved and tensed due to the fluorescent beads completely blocking all plasmin binding. (B) The potential digestive outcomes of a ridge-cleaved fiber are shown. The uppermost fiber is cleaved by plasmin on the left ridge, resulting in a fiber that shows no further digestion (middle) or recoils and collapses (bottom). (C) Bundling and elongation are shown. Two neighboring fibers are cleaved on either ridge, these two fibers bundle and the local bead concentration is increased. This increase in local bead concentration protects the fiber from further digestion, but the fiber is elongated due to the lytic process having already begun; shown by plasmin molecules present inside the fluorescent bead coating.

One possible explanation for this effect, outlined in Fig 8A, is that at the higher concentrations of fluorescent beads there could be a sheathing effect caused by beads coating the external surface area of the cylindrical fiber. This could either sterically hinder plasmin binding to fibrin, or beads could alter the local electrostatic environment causing plasmin molecules to be attracted to or repelled by the beads. Plasmin and its precursor plasminogen bind specifically to sites located at the periphery of fibrin molecules, α chain residues 148–160 in the “D” region, but can also bind to free C-terminal lysines that form during the digestion process [23]. Given the number of binding sites along the fiber length, sheathing by beads would need to be cohesive and extensive to block so many potential interactions. This is consistent with our bead diffusion simulations, which indicate the beads bind all along the fiber length (Fig 7). Plasmin may also non-specifically bind beads, which could be tested in future studies.

Also shown in Fig 2 is the tendency for un-cleaved remaining fibers at high bead concentrations to sustain their inherent tension, whereas at low bead concentrations remaining fibers tended to elongate over time. Fibers were all tense prior to plasmin addition, presenting as taunt polymers. A representative time series of fiber elongation is show in in Fig 1. Elongation is described as the loss of inherent tension that is present in fibers after polymerization [12, 13]. The origin of the inherent tension in fibers is uncertain, although we previously estimated that fibers are stretched 23% beyond an equilibrium length due to this inherent tension [14], which was validated by a recent mathematical model [24]. The mechanism for why remaining fibers at high bead concentrations remain tense, while remaining fibers at lower bead concentrations (especially between 4.55x10^9^ beads/μL and 1.52x10^9^ beads/μL) often elongate could also be explained by the proposed sheathing mechanism. High bead concentrations would block plasmin binding and result in little degradation and loss of tension, while lower bead concentrations could result in small gaps in the sheath where plasmin molecules could begin the lytic process. Presumably, elongated fibers are partially digested. Plasmin digests fibrin in a specific sequence, beginning with the unstructured followed by the coiled-coil region [23]. Perhaps elongated fibers have their αC regions digested, but not the coiled coil parts of the molecules. The sticky nature of the carboxylate-coated beads may maintain the longitudinal structural integrity of labeled fibers in this partially digested state. This would explain why elongation peaks at bead concentrations between 1.52x10^10^–4.55x10^9^ beads/μL, while at even lower concentrations most or all fibers are cleaved. Rimi and Helms (2022) also reported elongated fibers (“redistributed tension”, in their words), however, they only reported an elongation percentage (20%) for bead concentrations at 4.5 x10^8^ beads/μL [15], whereas in our experiments at bead concentrations that low, all the fibers were cleaved within 2 minutes. This difference may be due, in part, to the use of a higher thrombin concentration when polymerizing our samples, as previous work reported a correlation between thrombin concentration and cleavage, likely due to a difference in fiber diameter [12]. Of note, studies using fluorescent beads for labeling commonly report a “1:10,000” dilution [12, 14, 16, 17], corresponding to our bead concentration of 4.55x10^8^ beads/μL, but often don’t report how such a dilution was obtained. Based on our results, where most fibers lyse rapidly at such a low bead concentration, we suspect that bead concentrations may often be higher than what is reported due to errors in diluting to such a small value. In this study, careful serial dilutions were employed to ensure accuracy.

Fig 3 presents the rates of tensed and elongated remaining fibers at varying plasmin concentrations. During these tests, fluorescent beads were held at a 4.55x10^9^ beads/μL concentration. Plasmin concentration was varied between 0.7, 1.0, and 2.0 U/mL. At the lowest plasmin concentration, 0.7 U/mL, the highest number of remaining fibers was observed. As plasmin concentration increased, the number of cleaved fibers increased, and the percentage of remaining fibers fell but appears to be reaching a limit ~ 2 U/mL. Changing the plasmin concentration from 0.7 U/mL to 2 U/mL increased fiber cleavage percentage by ~15%. This suggests that bead concentration was a stronger determinant of fibrin cleavage than plasmin concentration. However, the increase in fiber cleavage as a function of plasmin concentration suggests that a higher number of plasmin molecules make it more likely for plasmin to find “chinks” in the bead “armor” that it can penetrate through to initiate lysis. While the percentage of remaining fibers decreased with increasing plasmin concentration, as expected, the ratio of tensed to elongated fibers remained roughly stagnant, supporting the idea that elongation is a function of bead concentration.

Bundling events in this study, Fig 4, were very common, with nearly one-fourth of all fibers that remained through the end of imaging having joined with one or more neighboring fibers. Bundling typically occurred when one fiber was cleaved and its remaining fragments bundled with nearby fibers. In some instances, where one fiber cleaved near a ridge, nearly the entire length of the cleaved fiber combined with a nearby fiber, creating a new fiber of similar length, but nearly twice as wide. Fibers that bundled were highly likely to elongate, with ~ 75% of bundled fibers displaying elongation. When two (or more) fibers bundle, the local bead concentration is increased [14], which likely helps inhibit new plasmin molecules from reaching the newly formed fiber (as demonstrated in Fig 8C).

When specifically looking at bundling in the bead concentration experiments, Fig 4B, a non-monotonic distribution is shown. At the highest bead concentration, 4.55x10^10^ beads/μL, very few fibers cleaved, resulting in nearly no fibers bundling. At concentrations of 1.52x10^10^ and 4.55x10^9^ beads/μL, a larger percentage of fibers elongated without bundling, but at 4.55x10^9^ beads/μL roughly equal amounts elongated after bundling and elongated without bundling. At fluorescent bead concentrations lower than 4.55x10^9^ beads/μL a decreasing number of uncleaved fibers left fewer fibers to elongate or bundle; however, those that did remain uncleaved tended to do so because of bundling, and those that bundled were likely to elongate. Statistical analysis demonstrated that the number of fibers that elongated without bundling was independent of bead concentration, while the number of fibers that bundled and elongated did depend on bead concentration. The results of Fig 4C showed that bundling remained a relatively constant phenomenon across all tested plasmin concentrations. At a plasmin concentration of 2 U/mL there were very few fibers that elongated without bundling, suggesting that when fibers were cleaved more rapidly, elongation did not have time to occur.

It is important to note that our experimental setup was specifically designed to isolate individual fibers. Thus, when a bundling event did occur, the fragments of one cleaved fiber often moved a distance of several microns before encountering another fiber to bundle with. In previous experiments investigating the lysis of 2-D dense networks of fibers that had also been labeled by beads, 30 ± 17% of fibers were observed to be bundled [14]. This agrees with the 27% of fibers bundled in our experiments even though we considered more sparsely situated fibers. In control experiments, where the fibers were unlabeled by beads, only 3% of fibers bundled, and most of the fibers that were observed to bundle were in areas where neighboring fibers were separated by less than 1 μm. This seems to suggest that beads increase the probability of bundling, even when fibers are separated by longer distances. However, the bundling observed in higher density areas of the control experiments suggests that in locations of high fiber density, fiber bundling occurs regardless of whether beads are present. These observations agree with another recent report that found that bead-labeled fibers in 3-D gels formed aggregates that were resistant to further degradation [15]. Previously it has been shown that bundling can aid the fibrinolytic process by means of network clearance, as plasmin does not have to individually digest fibers constituting a bundled network [14]. Conversely, here it is shown that in terms of individual bead-labeled fibers rather than networks, bundling has the opposite effect and increases the structural integrity of the fiber resulting from a bundling event. Thus, it appears that bundling plays competing roles during fibrinolysis.

With varying bead concentration there is also a difference in the location along a fiber in which cleavage occurred (Fig 5A). As bead concentration increased there was an increase in the percentage of fibers that cleaved along the length of the fiber, away from the ridge. These fibers that cleaved along their lengths recoiled in two directions and digested away as expected [10]. At the highest fluorescent bead concentration tested, 4.55x10^10^ beads/μL, there is an even split between fibers that cleaved at the ridge and cleaved along their length, although it should be noted that there were very few fibers cleaved at all at this concentration. Interestingly, using the same ridge-based experimental setup, for fibers that weren’t labeled by beads, 39% (179/455) cleaved at the ridge, while 61% (276/455) cleaved somewhere along the fiber length.

This result is somewhat surprising as intuition would suggest that as the beads become more dilute, the fiber cleavage sites should behave more like unlabeled fibers, but this was not the case. This effect could possibly be explained under the sheathing hypothesis by the assumption that beads binding in the complex angles formed between the fibers and the ridges is unfavorable, so at lower concentrations there are few beads near the ridges that would block plasmin from binding to fibrin, making those sites particularly susceptible to cleavage. At higher concentrations beads nearly completely cover the fibers, so any “exposed” plasmin binding site would be randomly located anywhere along the fiber and thus the location of fiber cleavage is more heterogeneous. The simulation results in Fig 7 support the idea that binding of beads to the fiber locations abutting the ridges is unfavorable. Since beads take longer and bind with less frequency to those locations, the fibrin at the ridge connection would be more available for plasmin binding and cleavage compared to locations along the length of the fiber.

Fibers that cleaved along their length typically had the two new fiber halves recoil backwards and collapse back onto the ridge. However, this was not always the case for fibers that cleaved at their connection to one of the ridges. When these fibers pulled away from the ridge, there were two observed outcomes: (1) the recoil and collapse/digestion of one long fiber segment, or (2) the long fiber segment showed no signs of further digestion (see Fig 5B). Fiber segments that showed no collapse or further digestion spent the remainder of data collection with the cleaved end loosely floating around in the buffer-plasmin solution, while the other end remained fixed to the ridge (see S4 Movie). These two outcomes occurred in roughly equal proportion to each other, independent of bead concentration (Fig 5B). This could be explained by considering the flexural rigidity of the fibers. Fibers covered in beads are likely stiffer than ones with fewer beads, and therefore the fibers are less likely to collapse backwards if bead concentrations are high. The recent observation of undigestible fibrin aggregates during the lysis of bead-labeled 3-D fibrin gels may arise from similar mechanisms to those undergirding our observation of undigestible fiber segments [15]. In both instances, the loss of tension after fiber cleavage may compress the spacing between beads, hindering the further digestion of the fibrin fragments/aggregates.

Bead concentration played a major role in the length of time for plasmin to cleave a fiber. As shown in Fig 6, high bead concentrations resulted in the prolongation of cleavage of an average fiber contained in each sample. When bead concentrations were reduced, the amount of time that passed before cleavage of the average fiber was also reduced. These results are consistent with the proposed sheathing mechanism (Fig 8A), as at high bead concentrations there would be fewer accessible binding sites for plasmin, resulting in a time delay dependent on how long it takes for a plasmin molecule to find a site to bind and begin digestion.

Our data provides some evidence that elongation is a function of light dosage, however statistical analysis was not indicative that this difference in means was significant (see Fig 2B). Light dosage tests indicated that leaving the shutter open during imaging resulted in 150-fold more light delivered to the sample. We hypothesize that large amounts of light could potentially deform the beads, leading to enhanced sheathing, and this could be tested in future experiments.

## Conclusions

Our results have implications for future fibrinolysis studies. This study has highlighted the potential pitfalls of utilizing fluorescence labeling in a system in which beads do not exist under physiological conditions. First, the addition of fluorescent beads to a fibrin network altered how single fibers, and thus the network, digested, with roughly one-third of all fibers observed persisting through digestion and elongating. Second, the concentration of fluorescent beads played a crucial role in single fiber degradation, with almost 100% of fibers labeled with the highest bead concentration tested not cleaving, and all fibers cleaving at the lowest bead concentration tested. Third, bead concentration correlated with the cleavage location, with low bead concentrations strongly favoring cleavage locations near the stamped ridges of optical adhesive. Lastly, fluorescent bead concentration altered the amount of time for cleavage to occur, with high bead concentrations resulting in longer cleavage times. These results have arisen from the testing of one brand, size, type, and corresponding wavelength of fluorescent bead, although it is likely our results would expand to other fluorospheres, as has been recently reported [15]. Furthermore, the results described here reflect experimental microscopy assays conducted at room temperature. The observed results may differ if an experimental setup attempted to mirror biological conditions such as human body temperatures, as temperature impacts molecular diffusion to a great extent. Our results suggest that fluorescent microspheres should, in general, not be used during fibrinolysis experiments, and if they must be used, should be used at very low concentrations.

## Supporting information

S1 File Supplemental document. This supplemental file contains plots and statistical analysis referred to in the main text.
(DOCX)

S2 File Source code for bead diffusion simulations. This code simulates the 2D diffusion of beads in a rectangular domain. It was used to generate the data in Fig 7 and the S5 Movie.
(M) Click here for additional data file.

S1 Movie Recoil and collapse movie: Movie of a bead labeled fiber (4.55x10^9^ beads/μL) displaying the expected recoil and collapse lytic behavior of fibrin fibers not labeled with fluorescent beads. (AVI)

S2 Movie Elongation movie: Movie of a bead labeled fiber (4.55x10^9^ beads/μL) displaying a loss of initial tension over time without cleaving. Note the taunt fiber structure in the beginning of movie (0 s) compared to the loose, “elongated”, structure present at the end of movie (60 min).
(AVI)

S3 Movie Bundling movie: Movie of two bead labeled fibers (4.55x10^9^ beads/μL) that bundle together after an initial cleavage event. Note that after bundling the newly formed fiber persists throughout the remainder of image acquisition (60 min).
(AVI)

S4 Movie Cleavage with no collapse movie: Movie of a bead labeled fiber (4.55x10^9^ beads/μL) that cleaves at the ridge connection, but shows no signs of further digestion, rather than collapsing as expected. (AVI)

S5 Movie Visualization of simulation with 100 beads. Beads (blue circles) diffuse in a 2 micron by 2 micron domain with reflecting boundary conditions on the top, left, and right boundaries. The bottom boundary (at y = 0) corresponds to the fibrin fiber. When a bead diffuses to a position on this boundary, it "binds" to the fiber at that location and remains there for all time. Each frame of the movie is 100 timesteps after the frame preceding it. The time step for this simulation was 0.000001 s. Beads, which have diameter 24 nm, are not drawn to scale.
(MP4)
