# Supplementary material for: Fluorescent microspheres can affect in vitro fibrinolytic outcomes
Source: PLoS One. 2023 Apr 7;18(4):e0284163. doi: 10.1371/journal.pone.0284163 (PMC10081780; doi:10.1371/journal.pone.0284163)
Supplement: S1 File — This supplemental file contains plots and statistical analysis referred to in the main text. (DOCX) [file pone.0284163.s001.docx]

Supporting Information

**Fluorescent microspheres can affect *in vitro* fibrinolytic outcomes**Ethan G. Stoll^1^, Sean J. Cone^2^, Spencer R. Lynch^1^, Andrew T. Fuquay^3^, Brittany E. Bannish^4^, Nathan E. Hudson^1^

^1^Dept. Physics, East Carolina University, Greenville, NC, USA.

^2^Department of Biological Sciences, North Carolina State University, Raleigh, NC 27606

^3^Medical Physics Graduate Program, Duke University; DUMC 2729, 2424 Erwin Rd Suite 101, Durham, NC 27705, USA

^4^Dept. Mathematics and Statistics, University of Central Oklahoma, Edmond, OK, USA.

We perform linear regression on the data from main text figures 4B, 4C, and 5B to explore relationships between percent fibers remaining and bead or plasmin concentration. Data in the main text is presented as total fiber count, but here we depict the independent trials (usually 5 per experimental condition).

## Transformation for regression

Since bead concentration varies by orders of magnitude, we compute regressions for main text Figures 4B and 5B with functions of the form

$$\text{percent}=a+b\times\left( \text{median-centered}\log_{10}\left( \text{bead concentration} \right) \right).$$

For Figure 4C, we use a function of the form

$$\text{percent}=a+b\times\left( \text{median-centered plasmin concentration} \right).$$

## Supplemental information for main text Figure 4B

Here we perform linear regression on the data from main text Figure 4B to explore the relationship between percent fibers remaining and bead concentration (Figure S1). Variables are centered around the median bead concentration ($4.55\times{10}^{9}$); thus, intercept estimates obtained from linear regression correspond to the value at median concentration. In other words, regression corresponds to a line in point-slope form where the point is located at the median bead concentration. Slopes represent percent per log concentration.

The intercept estimates are all significantly different from zero: 50.55% for percent remaining due to bundling ($p=2.24\times{10}^{-7}$, red), 44.83% for percent elongated and bundled ($p=4.46\times{10}^{-6}$, blue), and 16.64% for percent elongated without bundling ($p=0.0093$, yellow). Slopes reflect the effect of $\log10\left( \text{bead concentration} \right)$ and are significantly different from zero for two measures: $-57.9$ for percent remaining due to bundling ($p=3.59\times{10}^{-6}$, red), and $-52.9$ for percent elongated and bundled ($p=0.0003$, blue). The slope is not significantly different from zero for percent elongated without bundling (yellow): $-1.91$ ($p=0.8452$). This suggests that the percent elongated without bundling is largely independent of bead concentration, while the other two measures, which both involve bundling, significantly depend on bead concentration.


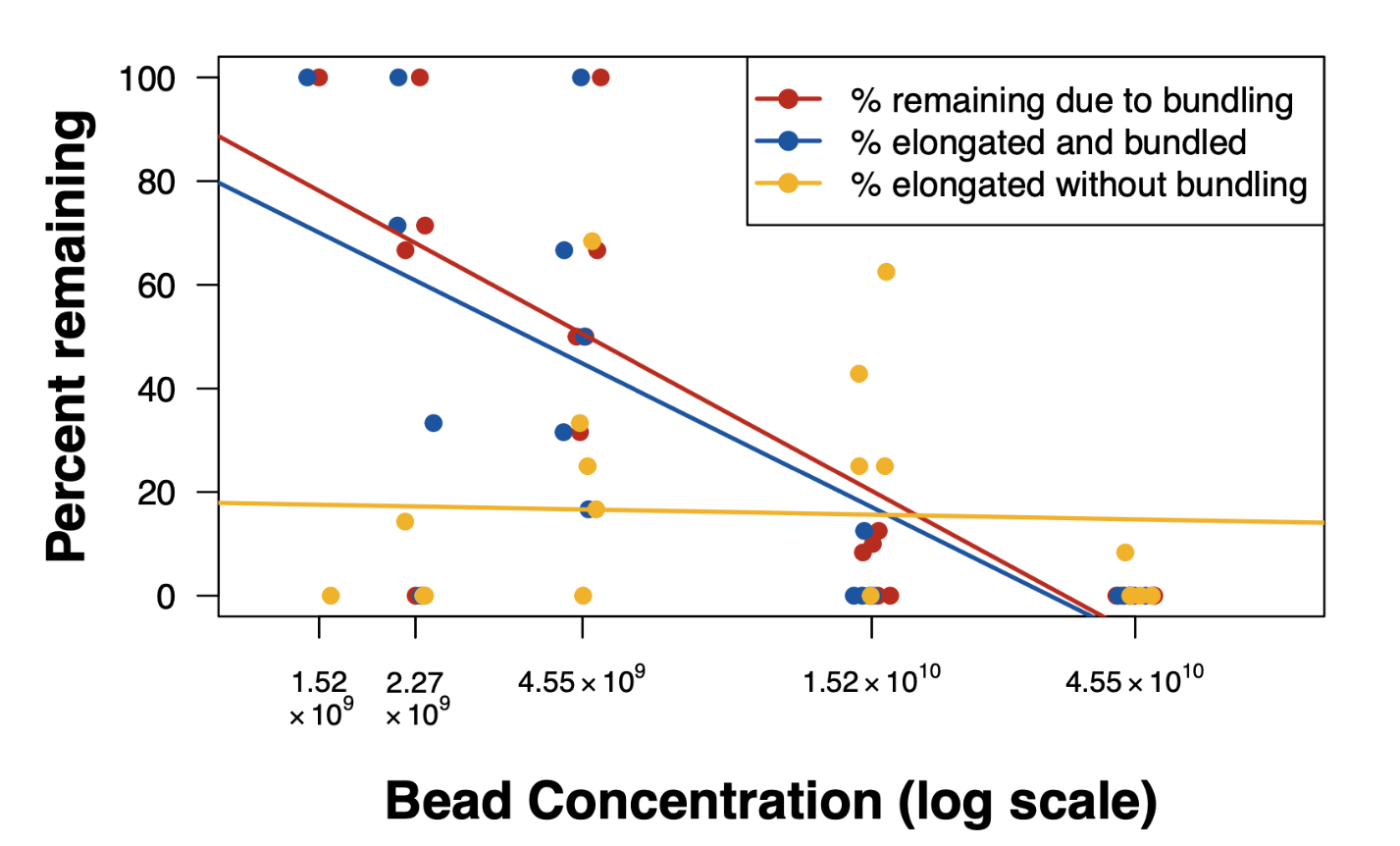


## Figure S1. Linear regression for Fig. 4B data. Plot of precent fibers remaining due to bundling (red), elongation with bundling (blue), and elongation without bundling (yellow) as a function of bead concentration. Lines represent the linear regressions. Note that horizontal scatter within a given concentration is for illustration purposes only.

## Supplemental information for main text Figure 4C

Here we perform linear regression on the data from main text Figure 4C to explore the relationship between percent fibers remaining and plasmin concentration (Figure S2). Intercepts reflect percent remaining at median plasmin concentration ($1.0\text{U per mL}$), while slopes represent percent per concentration.

All intercepts are significantly different from zero: 50.55% for percent remaining due to bundling ($p=2.04\times{10}^{-6}$, red), 39.35% for percent elongated and bundled ($p=0.0002$, blue), and 28.85% for percent elongated without bundling ($p=0.00106$, yellow). Slopes reflect effect of plasmin concentration and are not significantly different from zero for the first two measures: $11.89$ for percent remaining due to bundling ($p=0.2738$, red), and $3.80$ for percent elongated and bundled ($p=0.7687$, blue). The slope is significantly different from zero for percent elongated without bundling (yellow): $-0.2639$ ($p=0.0379$). This suggests that the percent elongated without bundling is dependent on plasmin concentration, but the other two measures (which both involve bundling) are not.


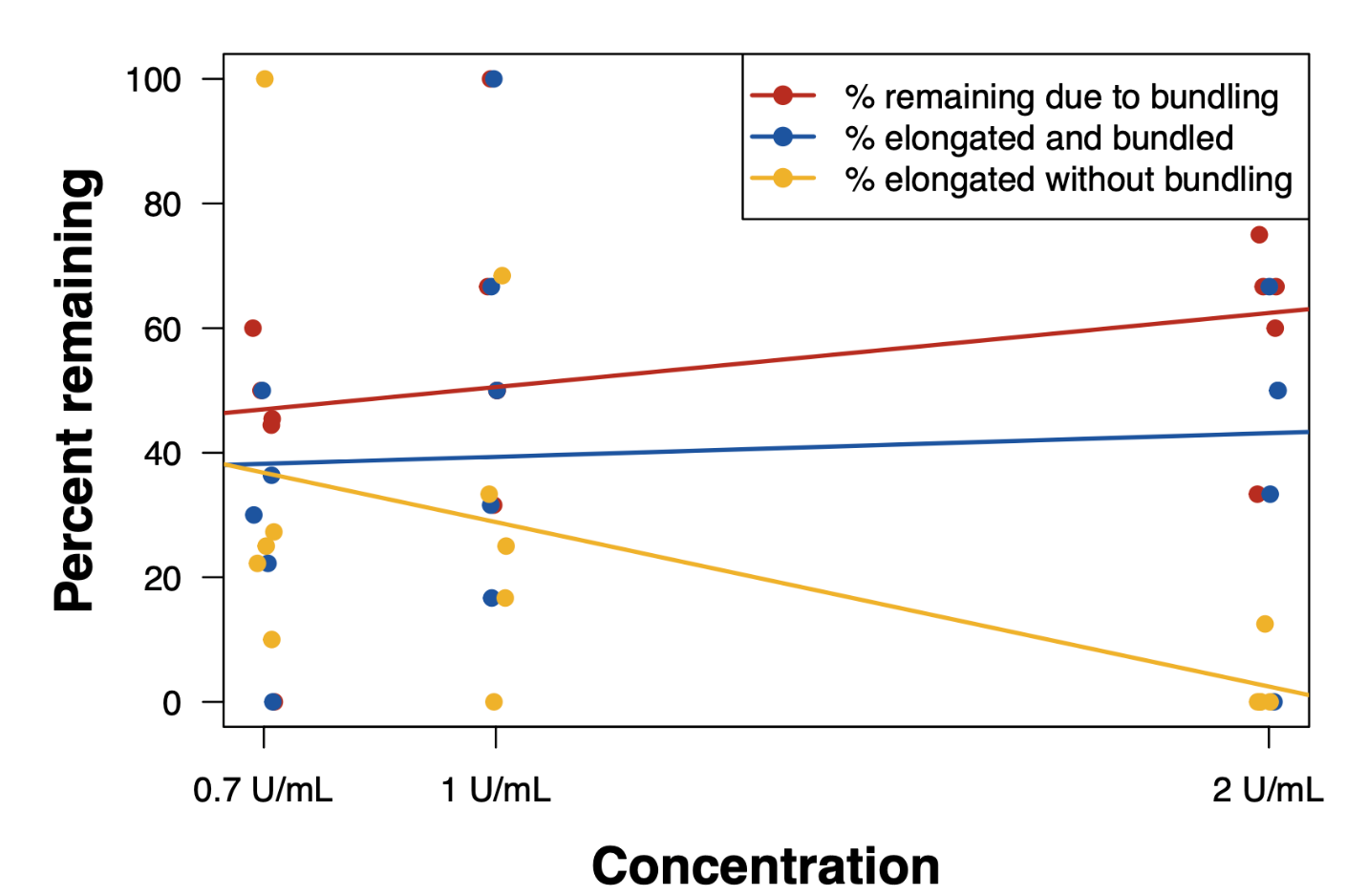


## Figure S2. Linear regression for Fig. 4C data. Plot of precent fibers remaining due to bundling (red), elongation with bundling (blue), and elongation without bundling (yellow) as a function of plasmin concentration. Lines represent the linear regressions. Note that horizontal scatter within a given concentration is for illustration purposes only.

## Supplemental information for main text Figure 5B

Here we perform linear regression on the data from main text Figure 5B to explore the relationship between digestive outcomes and bead concentration (Figure S3). Variables are centered around the median bead concentration ($4.55\times{10}^{9}$); thus, intercept estimates obtained from linear regression correspond to the value at median concentration. Slopes represent percent per log concentration.

The intercept estimates are all significantly different from zero: 77.26% for ridge cleaved fibers ($p=1.82\times{10}^{-13}$, blue), 45.33% for recoiled and collapsed fibers ($p=4.6\times{10}^{-8}$, red), and 45.31% for fibers with no further digestion ($p=1.2\times{10}^{-6}$, yellow). Slopes reflect the effect of $\log10\left( \text{bead concentration} \right)$ and are significantly different from zero for two measures: $-48.41$ for ridge cleaved fibers ($p=3.41\times{10}^{-5}$, blue), and $-33.67$ for recoiled and collapsed fibers ($p=0.00367$, red). The slope is not significantly different from zero for fibers with no further digestion (yellow): $-12.18$ ($p=0.348$). This suggests that the fibers with no further digestion are independent of bead concentration, while the other two fiber types (ridge cleaved and recoiled and collapsed) significantly depend on bead concentration.


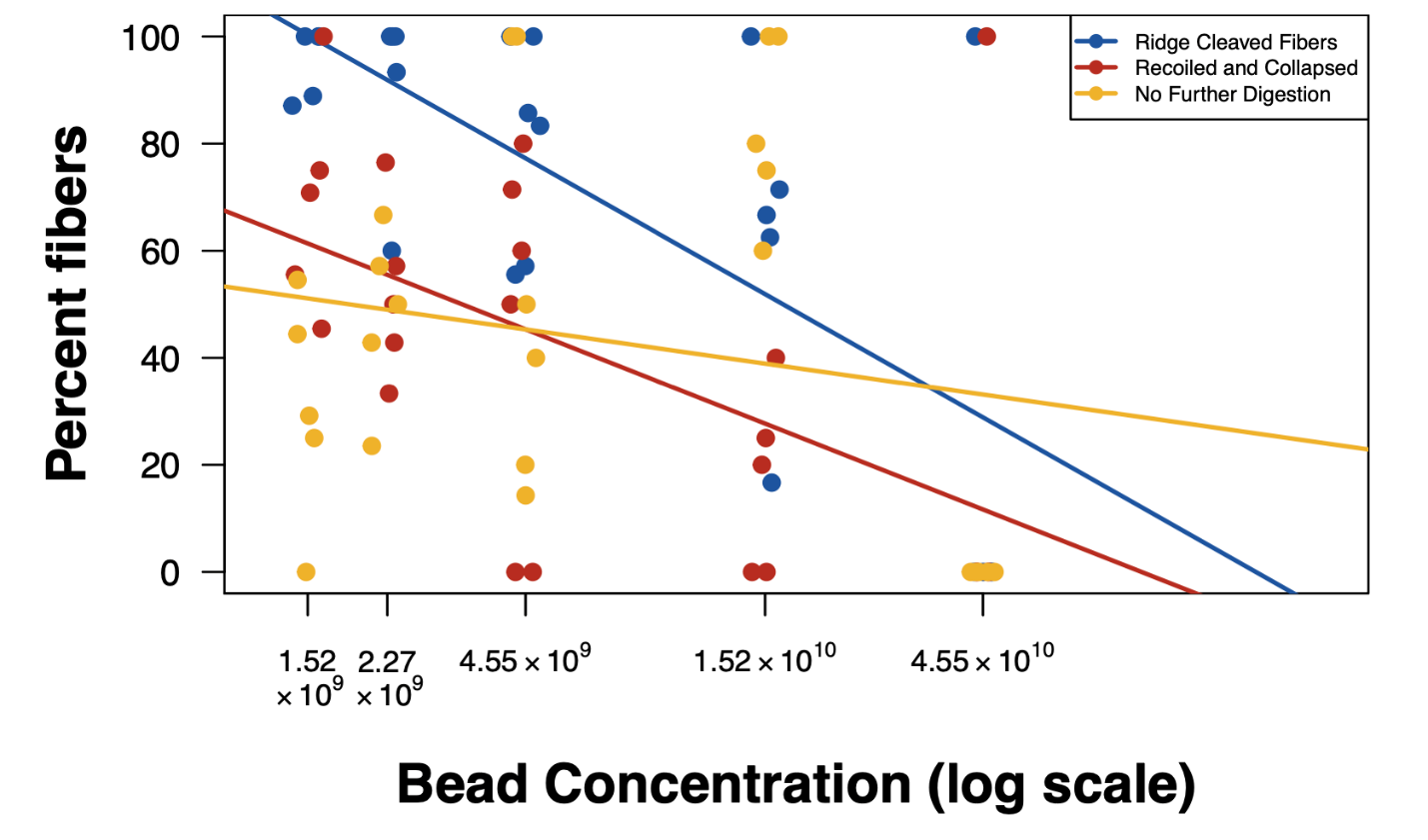


## Figure S3. Linear regression for Fig. 5B data. Plot of percent fibers remaining for each digestive outcome (ridge cleaved – blue, recoiled and collapsed – red, no further digestion – yellow) as a function of bead concentration. Lines represent the linear regressions. Note that horizontal scatter within a given concentration is for illustration purposes only.
